# Supplementary material for: FastqPuri: high-performance preprocessing of RNA-seq data
Source: BMC Bioinformatics. 2019 May 3;20:226. doi: 10.1186/s12859-019-2799-0 (PMC6500068; doi:10.1186/s12859-019-2799-0)
Supplement: Supplementary file 2 — Archive of FastqPuri. Archive containing all files needed to install and run FastqPuri v1.0.6. Date stamp March 22, 2019. (GZ 47,819 kb) [file 12859_2019_2799_MOESM2_ESM.gz › FastqPuri-1.0.6/html/globals_vars.html]

FastqPuri: File Members


FastqPuri


### - a -

- albw0
  : adapters.c
- albw1
  : adapters.c
- alfw0
  : adapters.c
- alfw1
  : adapters.c
- alloc\_mem
  : makeTree.c
  , makeBloom.c
  , fa\_read.c
  , bloom.c
  , trimFilterDS.c
  , trimFilter.c
  , tree.c

### - b -

- bitMask
  : bloom.c
- bw\_1B
  : trimDS.c
  , Lmer.c
  , adapters.c

### - f -

- fw0
  : bloom.c
- fw\_1B
  : trimDS.c
  , Lmer.c
  , adapters.c

### - n -

- Nencode
  : Lmer.c
  , trim.c

### - p -

- par\_MB
  : init\_makeBloom.c
  , makeBloom.c
- par\_MT
  : makeTree.c
  , init\_makeTree.c
- par\_QR
  : stats\_info.c
  , init\_Qreport.c
  , Qreport.c
  , Rcommand\_Qreport.c
- par\_SR
  : init\_Sreport.c
  , Rcommand\_Sreport.c
  , Sreport.c
- par\_TF
  : init\_trimFilter.c
  , trimDS.c
  , trim.c
  , init\_trimFilterDS.c
  , trimFilterDS.c
  , trimFilter.c


---

Generated on Mon Mar 19 2018 23:42:01 for FastqPuri by  

 1.8.14
